# Identification of novel therapeutic targets for chronic kidney disease and kidney function by integrating multi-omics proteome with transcriptome

**DOI:** 10.1186/s13073-024-01356-x

**Published:** 2024-06-19

**Authors:** Shucheng Si, Hongyan Liu, Lu Xu, Siyan Zhan

**Affiliations:** 1 Research Center of Clinical Epidemiology, Peking University Third Hospital, Beijing, 100191 China; 2 Peking University Health Science Center, Beijing, 100191 China; 3 Key Laboratory of Epidemiology of Major Diseases (Peking University), Ministry of Education, 38 Xueyuan Road, Haidian District, Beijing, 100191 China; 4 Department of Epidemiology and Biostatistics, School of Public Health, Peking University, Beijing, 100191 China; 5 Institute for Artificial Intelligence, Peking University, Beijing, 100871 China

**Keywords:** Chronic kidney disease, Kidney function, Mendelian randomization, Proteome, Transcriptome, Therapeutic targets

## Abstract

**Background:**

Chronic kidney disease (CKD) is a progressive disease for which there is no effective cure. We aimed to identify potential drug targets for CKD and kidney function by integrating plasma proteome and transcriptome.

**Methods:**

We designed a comprehensive analysis pipeline involving two-sample Mendelian randomization (MR) (for proteins), summary-based MR (SMR) (for mRNA), and colocalization (for coding genes) to identify potential multi-omics biomarkers for CKD and combined the protein–protein interaction, Gene Ontology (GO), and single-cell annotation to explore the potential biological roles. The outcomes included CKD, extensive kidney function phenotypes, and different CKD clinical types (IgA nephropathy, chronic glomerulonephritis, chronic tubulointerstitial nephritis, membranous nephropathy, nephrotic syndrome, and diabetic nephropathy).

**Results:**

Leveraging pQTLs of 3032 proteins from 3 large-scale GWASs and corresponding blood- and tissue-specific eQTLs, we identified 32 proteins associated with CKD, which were validated across diverse CKD datasets, kidney function indicators, and clinical types. Notably, 12 proteins with prior MR support, including fibroblast growth factor 5 (FGF5), isopentenyl-diphosphate delta-isomerase 2 (IDI2), inhibin beta C chain (INHBC), butyrophilin subfamily 3 member A2 (BTN3A2), BTN3A3, uromodulin (UMOD), complement component 4A (C4a), C4b, centrosomal protein of 170 kDa (CEP170), serologically defined colon cancer antigen 8 (SDCCAG8), MHC class I polypeptide-related sequence B (MICB), and liver-expressed antimicrobial peptide 2 (LEAP2), were confirmed. To our knowledge, 20 novel causal proteins have not been previously reported. Five novel proteins, namely, GCKR (OR 1.17, 95% CI 1.10–1.24), IGFBP-5 (OR 0.43, 95% CI 0.29–0.62), sRAGE (OR 1.14, 95% CI 1.07–1.22), GNPTG (OR 0.90, 95% CI 0.86–0.95), and YOD1 (OR 1.39, 95% CI 1.18–1.64,) passed the MR, SMR, and colocalization analysis. The other 15 proteins were also candidate targets (GATM, AIF1L, DQA2, PFKFB2, NFATC1, activin AC, Apo A-IV, MFAP4, DJC10, C2CD2L, TCEA2, HLA-E, PLD3, AIF1, and GMPR1). These proteins interact with each other, and their coding genes were mainly enrichment in immunity-related pathways or presented specificity across tissues, kidney-related tissue cells, and kidney single cells.

**Conclusions:**

Our integrated analysis of plasma proteome and transcriptome data identifies 32 potential therapeutic targets for CKD, kidney function, and specific CKD clinical types, offering potential targets for the development of novel immunotherapies, combination therapies, or targeted interventions.

**Supplementary Information:**

The online version contains supplementary material available at 10.1186/s13073-024-01356-x.

## Background

Chronic kidney disease (CKD) is a progressive disease characterized by structural and functional damage which affects approximately 10% of the world’s population [1, 2]. People with CKD face high risks of many adverse outcomes, including the need for kidney replacement therapy, cardiovascular events, and death [1]. However, such a serious and widespread disease has no effective cure in clinical practice, and novel strategies were required to prolong kidney and patient survival without dialysis and kidney transplantation [3]. A deeper identification and understanding of the biomarkers involved in the CKD biological pathways is essential for identifying potential treatment targets.

In biological mechanisms, the fundamental flow of information in biological systems is from DNA (genome) to RNA (transcriptome) to proteins (proteome) [4]. Large-scale genome-wide association studies (GWAS) have identified hundreds of loci that are associated with CKD and kidney function [5–7]. Nonetheless, these loci, which are upstream of the biological mechanisms, are a long way from being useful for therapeutic targets. The current blood proteome permits high-throughput analysis and identification of potential targets, including those enriched for CKD or its risk factors [8]. Meanwhile, Mendelian randomization (MR) is an approach that uses genetic variation as instrumental variables (IVs) to infer causal relationships between exposures and outcomes. Before investigating dedicated animal models or randomized trials, the MR method with quantitative trait locus (QTLs) as IVs can add evidence for causal inference in CKD proteomics research [8]. For example, some recent studies have explored proteomics for CKD progression and estimated glomerular filtration rate regulation (eGFR) regulation by specific cohorts or Mendelian randomization-based methods [9–11]. Schlosser et al. nominated determinants of kidney filtration (eGFR, blood urea nitrogen) and kidney damage (albuminuria) by transcriptome and proteome-wide association studies [12]. Some studies have also provided evidence of the effects of specific proteins, such as FGF21, and specific genes, such as ALCY and CVD- and inflammation-related proteins, on changes in kidney function [13–15]. Nonetheless, a comprehensive understanding of the integration of large-scale transcriptomic and proteomic data across diverse CKD- and kidney function-related phenotypes, which is crucial for confirming prior findings, discovering novel biomarkers, and revealing new roles, remains lacking. This gap may be attributed to factors such as the availability of high-quality data with larger sample sizes in transcriptomic and proteomic GWASs, tissue-specific transcriptomic variations, and insufficient inclusion of CKD phenotypes. Therefore, systematic research is necessary to overcome these limitations, enhancing our understanding of multi-omics biomarkers of CKD and kidney function and facilitating the identification of therapeutic targets through well-designed analytical frameworks.

With the development of aptamer-based and immunoassay-based platforms, including SomaScan and Olink, for more than ~ 1000 to 7000 proteins, large-scale GWAS datasets for the plasma proteome involving large-scale samples, such as studies on 35,559 Icelanders, 10,708 Fenland, and 54,306 UK Biobank participants, have been released [8, 16–18]. These protein biomarkers could be well matched with coding genes in available GWAS datasets for transcriptomes, such as eQTLGen and GTEx [19, 20]. The integration of genomics with the transcriptome and proteome may greatly contribute to testing whether selected biomarkers are involved in causal pathways. We hypothesized that an ideal target of CKD may follow the flow of biological mechanisms, while the efforts to integrate analyses of multiple proteome and transcriptome could deepen our understanding of therapeutic approaches to kidney disease.

In this study, we integrated the top 3 largest GWAS for more than 3000 proteins with available pQTLs, the corresponding largest GWASs of blood and tissue-specific gene expression (mRNA), and extensive GWASs of CKD-related outcomes, including CKD, trans-ancestry CKD, kidney function, rapid kidney function decline, annualized relative change of kidney function, and some specific CKD clinical types. With a comprehensive analysis pipeline including proteome-wide MR, transcriptome-wide MR, colocalization analyses, protein–protein interaction (PPI), and gene enrichment analysis, this study aimed to identify the potential causal protein biomarkers and target genes that are druggable for future CKD treatment.

## Methods

### Study design

**Fig. 1:**
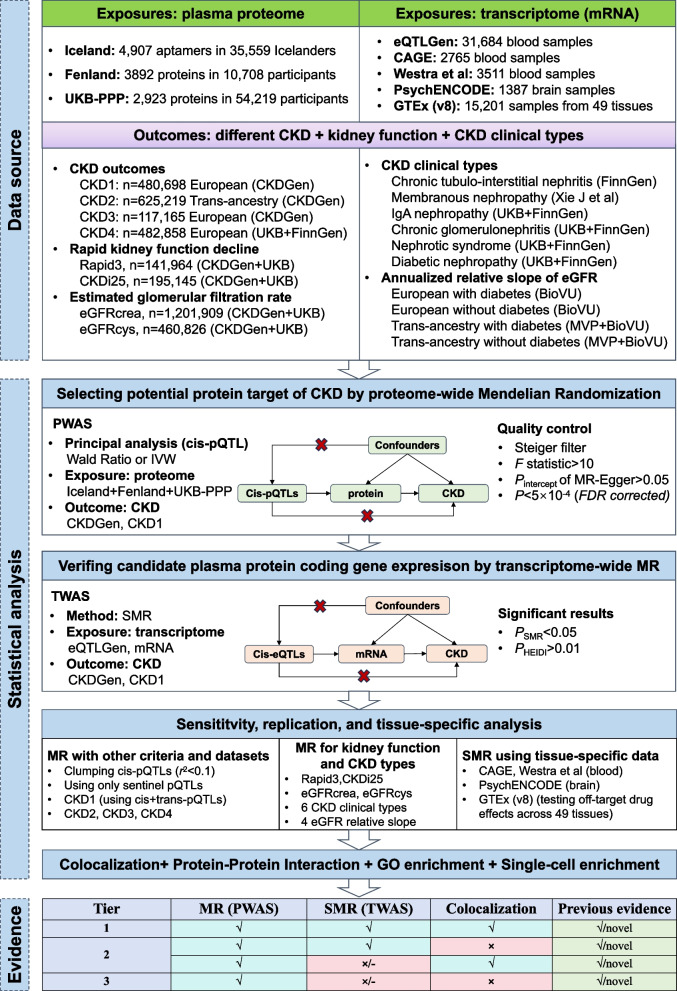
Overview of the study design. (1) The exposure summary data include three proteome-wide pQTL and five transcriptome-wide eQTL datasets. The outcome summary data include four CKD outcomes (data CKD1–4), two kidney function phenotypes (eGFRcrea + eGFRcys), two rapid kidney function decline phenotypes (Rapid3 + CKDi25), annualized relative slope change of eGFR in four populations, and six CKD clinical types. (2) The workflow of the statistical analysis included proteome-wide MR (PWAS), transcriptome-wide MR (TWAS), sensitivity, replication, tissue-specific analysis, colocalization analysis, protein–protein interaction analysis, GO enrichment analysis, and single-cell enrichment annotation. (3) the three evidence tiers were determined according to MR, SMR, and colocalization analysis and compared with previous evidence

The study design is shown in Fig. 1. We defined a comprehensive analysis pipeline: (1) to select potential protein targets of CKD from three large GWAS datasets by proteome-wide association study (PWAS) using the two-sample MR method; (2) to verify the expression of these candidate plasma protein-coding genes and to identify consistent associations by a transcriptome-wide association study (TWAS) using the summary-based MR (SMR) method; (3) to explore the roles of these candidate protein targets in trans-ancestry CKD, kidney function phenotypes, and different CKD clinical types by sensitivity and replication analysis; (4) to verify the shared coding gene loci of identified protein with CKD by colocalization analysis; (5) to explore the potential biological mechanism of putative protein targets by protein–protein interaction (PPI) and Gene Ontology (GO) enrichment analysis; and (6) to classify the evidence of this study by the results of MR, SMR, colocalization, and comparisons with previous evidence.

### Data sources of plasma proteome

As shown in Fig. 1, we acquired pQTLs associated with plasma proteins from the three largest independent GWASs—(1) 28,191 genetic associations (*P* < 1.8 × 10^−9^) for 4907 aptamers were identified in 35,559 Icelanders based on the SomaScan platform. The data are derived from two main projects: the Icelandic Cancer Project (ICP) (52% of participants) and various genetic programs at deCODE Genetics, Reykjavík, Iceland (48% of participants) [16]. These precalculated summary statistics used recursive conditional analysis to denote the most significant variant in each region (± 1 Mb) as the sentinel pQTL (*n* = 18,084) and the other variants as secondary pQTLs (*n* = 10,107) [16]. This GWAS replicated 83% of reported pQTLs in the INTERVAL study (based on SomaScan) and 64% of the pQTLs from the SCALLOP consortium (based on Olink) [16]. (2) A total of 10,674 genetic associations (*P* < 1.004 × 10^−11^) for 3892 plasma proteins were identified in 10,708 European-descent participants from Fenland using the SomaScan platform [17]. Conditional analysis was also utilized to detect sentinel (*n* = 8328) and secondary signals (*n* = 2346) for each genomic region identified by distance-based clumping with GCTA [17]. This GWAS replicated 61% of pQTLs using the Olink technique, with a higher proportion for cis-pQTLs (81.2%) [17]. (3) A total of 23,588 primary (sentinel) genetic associations (*P* < 1.7 × 10^−11^, clumping ± 1 Mb, *r*^2^ < 0.8) for 2923 proteins in 54,219 participants from the UK Biobank Pharma Proteomics Project (UKB-PPP) were identified using the Olink platform [18]. This GWAS replicated 84% of the previous pQTLs from antibody-based studies and replicated 38% of pQTLs from aptamer-based studies [18]. These pQTL summary statistics were obtained directly from the previous GWASs and therefore were not further adjusted for specific metrics such as eGFR.

According to these signals from the three GWASs, cis-pQTLs were defined as SNPs within 1 Mb from the gene encoding the protein, while the trans-pQTL exceeded 1 Mb from the gene encoding the protein. The details of these pQTLs are shown in Additional file 1: Fig. S1. A total of 3032 proteins (1439 in Iceland, 1563 in the UK Biobank, and 1399 in Fenland) (Additional file 1: Fig. S2) with available cis-pQTLs were utilized in subsequent analysis.

### Data sources of transcriptome

Gene expression data were sourced from the eQTLGen consortium, which provided us with a substantial sample size (*n* = 31,684) to identify SNPs associated with the expression of genes targeted by the corresponding plasma proteins [19]. In this study, we specifically focused on cis-eQTLs, ensuring the relevance of the genetic variation to gene expression changes. For replication, we also acquired 2 sets of gene expression data from the blood sample [CAGE (*n* = 2765) and Westra et al. (*n* = 3511)] and 1 set of data from the brain sample [PsychENCODE project (*n* = 1387)] [21–23]. We additionally acquired the tissue-specific cis-eQTLs from 49 tissues (*n* = 15,201) from the GTEx (v8) project [20] to explore the tissue-specific associations and potential off-target effects of drugs targeting genes. All of the gene expression datasets were publicly available and precalculated summary statistics. The eQTL data are represented as the effect of each additional allele on a 1-SD change in the gene expression level (mRNA).

### Data sources of chronic kidney disease

We acquired summary statistics for CKD from 3 large-scale genome-wide association meta-analyses. The principal dataset was the largest GWAS of CKD (defined as eGFRcrea < 60 mL min^−1^ per 1.73 m^2^) from CKDGen, which included 480,698 (41,395 cases) European ancestry participants across 23 studies (CKD1) and 625,219 (64,164 cases) trans-ancestry participants across 30 studies (CKD2) [5]. We also acquired additional GWAS data for CKD of European ancestry, which included 43 studies, for a total sample size of 117,165 (12,385 cases) (CKD3) [6]. Approximately 43.7% (*n* ≈ 51,171) of the samples from CKD3 participants overlapped with those from CKD2 participants (Additional file 2: Table S1). GWAS data from the UK Biobank plus FinnGen with 482,858 participants (8287 cases) for chronic renal failure were also included (CKD4) (UK Biobank endpoint: ICD10: N18 or phecode: 585.3; FinnGen endpoint: N14_CHRONKIDNEYDIS) [24].

### Data sources of kidney function

We included 3 GWAS summary statistics of different kidney function phenotypes. First, we used the largest glomerular filtration rate estimated from creatinine (eGFRcrea) and eGFRcys (cystatin-based eGFR) GWAS for direct kidney function measurement from the combined CKDGen and UK Biobank (*n* = 1,201,909 and 460,826, respectively) [7]. The 2 outcomes in this GWAS meta-analysis are log-transformed eGFRcrea and eGFRcys. Second, 2 phenotypes of rapid kidney function decline defined by eGFRcrea [≥ 3 mL/min/1.73 m^2^/year (“Rapid3,” 34,874 cases, 107,090 controls) and eGFRcrea decline ≥ 25% and eGFRcrea < 60 mL/min/1.73 m^2^ at follow-up among those with eGFRcrea ≥ 60 mL/min/1.73 m^2^ at baseline (“CKDi25”; 19,901 cases, 175,244 controls)] were acquired from a GWAS with 42 studies [25]. The 2 binary phenotypes represent the speed and proportion of eGFR decline, respectively. Third, annualized relative slope change of eGFR (interpreted as the percentage change in eGFR per year) was acquired from a study on Million Veteran Program (MVP) and Vanderbilt University Medical Center’s DNA biobank (BioVU) among participants with CKD stratified by ethnicity and diabetes status, including European participants with/without diabetes (*n* = 1642 and 5648, respectively) and trans-ancestry participants with/without diabetes (*n* = 46,424 and 70,446, respectively) [26].

### Data sources of CKD clinical types

The chronic tubulointerstitial nephritis data were acquired from the GWAS of the FinnGen consortium, which included 620 cases and 201,028 controls [27]. The data on membranous nephropathy were derived from a GWAS on European ancestry with 2150 cases and 5829 controls [28]. The IgA nephropathy (15,587 cases and 462,197 controls), chronic glomerulonephritis (566 cases and 475,255 controls), nephrotic syndrome (775 cases and 475,255 controls), and diabetic nephropathy (1032 cases and 451,248 controls) data were acquired from meta-analyses with the UK Biobank and FinnGen [24].

### Statistical analysis

#### Proteome-wide Mendelian randomization analysis

This study followed the STROBE-MR analysis guidelines. The potential protein targets for CKD were selected from three large plasma proteomics datasets by MR analysis. In principle analysis, only cis-pQTLs were utilized as IVs for each protein, while the outcome was from the CKDGen with European ancestry (CKD1). For proteins with only one cis-pQTL, the Wald ratio and the delta method were applied for estimating odds ratios (ORs) and corresponding confidence intervals (CIs) [29]. For proteins with multiple cis-pQTLs, estimators were acquired through the inverse variance weighted (IVW) method [30].

According to the causal graph of MR shown in Fig. 1, this method should satisfy three assumptions: (1) the IV is associated with the exposure, (2) the IV affects the outcome only through the exposure (lower red cross), and (3) the IV is not associated with the confounders (upper red cross). To satisfy these assumptions, several measures were applied. Testing for the intercept of MR‒Egger regression was performed to assess the existence of horizontal pleiotropy [31]. To further control potential reverse causality and pleiotropy, we used the Steiger filter to remove the pQTLs that explained more variance for CKD other than the corresponding protein. The IVs restricted to the cis-pQTLs and combined with colocalization analyses could reduce genetic confounding due to horizontal pleiotropy and linkage disequilibrium, respectively [12]. The *F*-statistic was calculated to assess the strength of the IVs. For pQTL *j*, the *F*-statistic can be approximated as *F*_*j*_ = γ̂_*j*_^2^/σ_*Xj*_^2^, where γ̂_*j*_ is the pQTL-protein association and σ_*Xj*_^2^ is the standard error of the association [32]. The heterogeneity of pQTLs was tested by *Q* statistics.

The MR estimators represented a per-SD increase in genetically predicted levels of circulating proteins on the risk of CKD. To address multiple testing, an *FDR*-corrected *P*-value (*q* < 0.05, approximate *P* < 5 × 10^−4^) was considered significant. Finally, the effects of a specific protein were meta-analyzed using the fixed-effect model if the protein was significant (*q* < 0.05) in any one of the three protein datasets, and estimators were available from more than one dataset. The corresponding results are reported as the “combined” effects.

#### Transcriptome-wide Mendelian randomization analysis

To further verify the detected plasma protein targets (*q* < 0.05), the summary-based MR (SMR) method [33] was utilized to evaluate the association between the corresponding protein-coding gene expression from the eQTLGen blood samples and the risk of CKD (data CKD1). The SMR approach selects the single most significantly associated eQTL SNP (located near the target gene) as an instrument. The default *P*-value for selecting the top associated eQTL was 5 × 10^−8^. The SMR tool also implements the heterogeneity in dependent instruments (HEIDI) test to assess whether the observed association between gene expression and outcome is due to a linkage scenario rather than the SNP affecting disease via gene expression regulation. A HEIDI test *P*-value < 0.01 was considered to indicate an association due to a linkage scenario. The main results are also presented as the ORs for disease per 1-SD change in gene expression.

#### Sensitivity, replication, and tissue-specific analysis

For IVs, we further clumped the cis-pQTLs by the “clump_data” function with the parameters clump_kb = 10,000 and clump_r2 = 0.01 to control the potential linkage disequilibrium. We also utilized only the sentinel (primary) cis-pQTLs for each protein for another sensitivity analysis. In addition, the combined cis- and trans-pQTLs were utilized as IVs to repeat the principal analysis. For outcomes, we used three other CKD data sources (data CKD2-4) to replicate our MR analysis. We also explored the associations of the identified proteins and two kidney function outcomes (eGFRcrea and eGFRcys), two rapid kidney function decline outcomes (Rapid3 and CKDi25), annualized relative slope change of eGFR in four populations, and six clinical types of CKD. For gene expression, we replicated our analysis with another two datasets with blood samples (CAGE and Westra et al.) and tissue-specific datasets (PsychENCODE and GTEx). The potential off-target effects of a drug targeting a gene were further assessed by determining whether these effects were contradictory across different tissues. There was no sample overlap in the principal MR analysis and all of the SMR analysis. The population of proteins from the UKB-PPP partly overlapped with the outcomes from the UK Biobank and was only utilized in replication analyses to verify the robustness of our findings.

#### Colocalization analysis

Colocalization analysis was applied to test whether the identified associations of proteins with CKD shared the same causal variant. The analysis was based on a Bayesian model with a posterior probability of five hypotheses (PPH): (1) no association with either trait (H_0_), (2) association with trait 1 only (H_1_), (3) association with trait 2 only (H_2_), (4) distinct causal variants associated with two traits (H_3_), and (5) same causal variant associated with both traits (H_4_) [34]. The “coloc.abf” algorithm was used with the default parameters (prior probability that a SNP is associated with trait 1: p1 = 1 × 10^−4^, with trait 2: p2 = 1 × 10^−4^, and with both traits p12 = 1 × 10^−5^). We defined the association between the identified protein and CKD as colocalization when the PPH_4_ > 0.8, while PPH_4_ > 0.5 indicated moderate colocalization.

#### Protein–protein interaction network, Gene Ontology enrichment analysis, single-cell enrichment annotation, and evidence from previous studies

To test the interactions of the identified proteins, we performed protein–protein interaction (PPI) network analysis for the proteins significantly associated with CKD (*q* < 0.05). All PPI analyses were conducted using the Search Tool for the Retrieval of Interacting Genes (STRING) database version 11.5 (https://string-db.org/), with the minimum required interaction score of 0.4 [35]. In addition, gene function annotation was performed for the identified protein-coding genes using biological function Gene Ontology (GO) enrichment analysis. GO enrichment analysis was used to analyze the biological significance of candidate genes, including biological process (BP), cellular component (CC), and molecular function (MF) enrichment; *q* < 0.05 was considered to indicate significant enrichment. Single-cell transcriptomic annotation for the 32 protein-coding genes was obtained from the Human Protein Atlas (proteinatlas.org), which provides the normalized protein transcripts per million reads for 76 cell types from 14 healthy tissue types [36]. The genes were enriched by RNA single cell type specificity, RNA tissue cell type specificity, and immune cell specificity. Then, whether the identified proteins and genes were druggable was determined through the previous study [37]. Finally, to explore whether our findings were reported by previous GWAS, transcriptome-wide MR, proteome-wide MR, or observational studies and whether the effects were consistent, we reviewed the related studies and compared the findings. The strategy of the review is described in the Additional file 1: Supplement Text.

All the statistical tests were two-tailed. The R software (version 4.3.1) with the TwoSampleMR [38], fdrtool [39], meta [40], coloc [34], clusterProfiler [41, 42], org.Hs.eg.db [43], and enrichplot [42] packages and the smr-1.3.1-win [33] software were used in this study.

## Results

### Putative plasma proteins on CKD

**Fig. 2:**
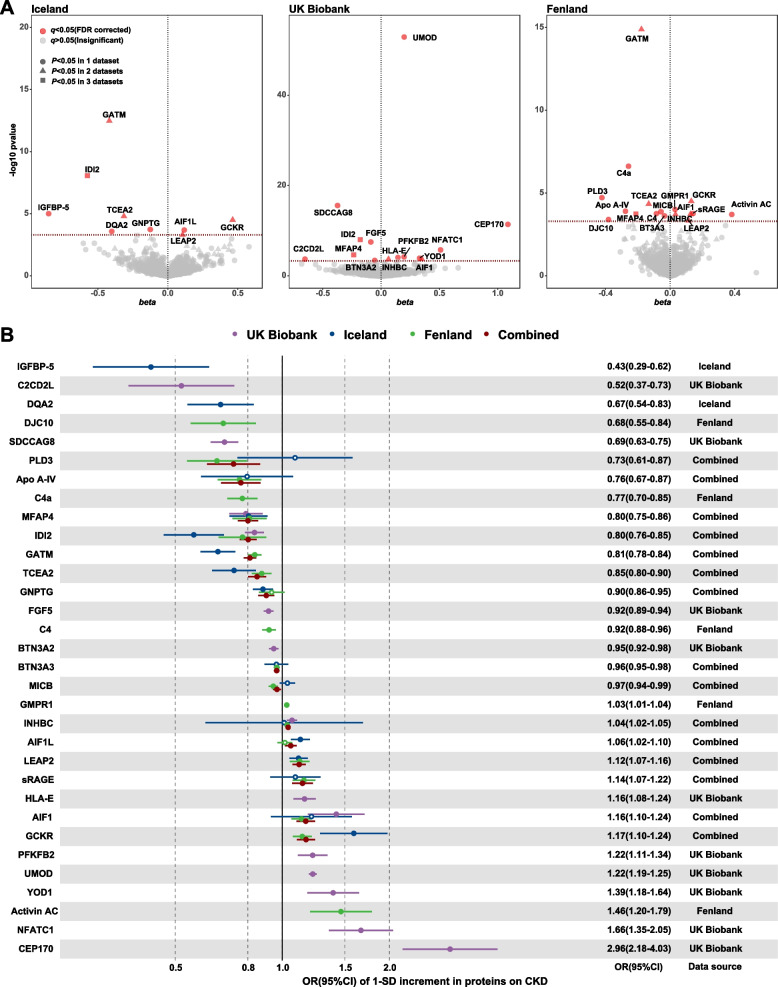
Associations of the 32 identified proteins with CKD. **A** Volcano plot of individual proteins associated with the primary CKD outcome across three data sources. The red line represented the threshold of FDR correction (*q* < 0.05), and the red point indicated significant proteins. **B** Forest plot of identified proteins in **A** that passed the FDR corrections for the risk of CKD. The ORs and 95% CIs of the significant proteins in any 1 dataset were reported. For proteins that were available from more than 1 dataset, the ORs and 95% CIs were combined by fixed effect meta-analysis and are shown as combined effects. Hollow dots represent *P* > 0.05, and solid dots represent *P* < 0.05. *Abbreviations*: IGFBP-5, insulin-like growth factor-binding protein 5; C2CD2L, C2 domain-containing protein 2-like; DQA2, HLA class II histocompatibility antigen, DQ alpha 2 chain; DJC10, DnaJ homolog subfamily C member 10; SDCCAG8, serologically defined colon cancer antigen 8; PLD3, phospholipase D3; Apo A-IV, apolipoprotein A-IV; C4a, complement component 4A; MFAP4, microfibril-associated glycoprotein 4; IDI2, isopentenyl-diphosphate delta-isomerase 2; GATM, glycine amidinotransferase, mitochondrial; TCEA2, transcription elongation factor A protein 2; GNPTG, *N*-acetylglucosamine-1-phosphotransferase subunit gamma; FGF5, fibroblast growth factor 5; C4, complement C4; BTN3A2, butyrophilin subfamily 3 member A2; BTNA3 (equal to BTN3A3), butyrophilin subfamily 3 member A3; MICB, MHC class I polypeptide-related sequence B; GMPR1, GMP reductase 1; INHBC, inhibin beta C chain; AIF1L, allograft inflammatory factor 1-like; LEAP2, liver-expressed antimicrobial peptide 2; sRAGE, advanced glycosylation end product-specific receptor, soluble; HLA-E, HLA class I histocompatibility antigen, alpha chain E; AIF1, allograft inflammatory factor 1; GCKR, glucokinase regulatory protein; PFKFB2, 6-phosphofructo-2-kinase/fructose-2,6-bisphosphatase 2; UMOD, uromodulin; YOD1, ubiquitin thioesterase OTU1; activin AC, inhibin beta A chain:inhibin beta C chain heterodimer; NFATC1, nuclear factor of activated T cells, cytoplasmic 1; CEP170, centrosomal protein of 170 kDa

The signals of significant proteins from 3 datasets on CKD are shown in Fig. 2A. After FDR correction, 32 proteins were significantly associated with CKD (*q* < 0.05), involving 9, 14, and 17 proteins from Iceland, UK Biobank, and Fenland, respectively. Among these proteins, IDI2 and MFAP4 were repeated in all 3 data sources, while GATM, TCEA2, INHBC, LEAP2, GCKR, INHBC, and AIF1 were repeated in 2 data sources. The *F*-statistics and MR-Egger test for the intercept are shown in Additional file 2: Tables S2-S3. In this study, all of the *F*-statistics were larger than 10, which was considered to indicate no weak IVs bias. The MR-Egger intercept test for pleiotropy was also satisfactory.

### Associations of putative proteins on CKD

The effects of 32 proteins were shown in Fig. 2B, 18 proteins were negatively associated with CKD and 14 proteins increased the CKD risk. MFAP4, IDI2, GATM, and TCEA2 were negatively associated with CKD in at least 2 datasets, where the corresponding combined ORs and 95% CIs were 0.80 (0.75–0.86), 0.80 (0.76–0.85), 0.81 (0.78–0.84), and 0.85 (0.80–0.90), respectively. In addition, INHBC, LEAP2, AIF1, and GCKR were positively associated with CKD, with ORs and 95% CIs of 1.04 (1.02–1.05), 1.12 (1.07–1.16), 1.16 (1.10–1.24), and 1.17 (1.10–1.24), respectively. The protein AIF1L in Fenland data and the protein sRAGE, INHBC, AIF1, and Apo A-IV in Iceland data presented significant heterogeneity tested by *Q* statistics (Additional file 2: Table S4). However, the effects of these proteins were not significant in the MR analysis (Fig. 2B) but had the same directions as the corresponding proteins from other data sources, which have limited influence on the overall results (the combined effects). In the sensitivity analysis with clumped pQTLs (*r*^2^ < 0.1), all 32 associations were replicated (Additional file 1: Fig. S3). When only the sentinel pQTLs were used, 31 of the 32 associations were replicated, except for AIF1L (Additional file 1: Fig. S4). In the sensitivity analysis with both cis- and trans-pQTLs, the results also presented the same direction, although some were not significant since the trans-pQTLs were more likely to be pleiotropy (Additional file 1: Fig. S5). The entire results for all 32 proteins (*q* < 0.05) in each data source are shown in Additional file 2: Table S5. The pQTL-exposure and pQTL-outcome associations are shown in Additional file 2: Table S6.

### Associations of the protein-coding gene expression on CKD

**Fig. 3:**
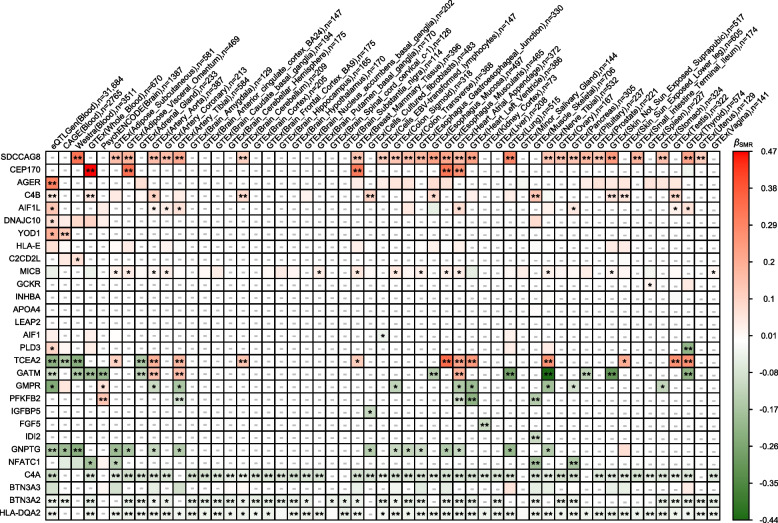
Heatmap of identified protein-coding genes associated with CKD. Heatmap of the effect of plasma and tissue-specific protein-coding gene expression on CKD risk for the identified proteins. The color represents the* β* estimators of SMR analysis, where green represents a decreased CKD risk and red represents an increased CKD risk for per-SD increased gene expression. *P < 0.05; **multiple tests,* P* < 0.05/29 (genes). The missing values marked with “-” represent the genes without effective eQTLs in the SMR analysis or failed in the HEIDI test

We mapped the 32 proteins to 29 coding genes. Figure 3 shows the SMR analysis results for 29 genes. Fourteen of 29 genes presented consistent results for CKD as the corresponding proteins. Among these genes, HLA-DQA2, BTN3A2, C4A, NFATC1, and GNPTG were associated with a decreased CKD risk in more than 1 blood sample or tissue-specific sample. In contrast, SDCCAG8, CEP170, AGER, C4B, AIF1L, DNAJC10, YOD1, CDCD2L, and MICB were significantly associated with increased CKD risk. In kidney cortex samples, the gene expression of FGF5, C4a, and HLA-DQA2 was negatively associated with CKD. The ORs and 95% CIs of the associations in the eQTLGen dataset are shown in Additional file 1: Fig. S6.

The genes TCEA2, GMPR, PLD3, GATM, and PFKFB2 presented different effects on CKD risk across blood or tissue-specific samples, which may reflect potential off-target effects. For example, TCEA2 gene expression in the blood and adrenal gland decreased the CKD risk, which was consistent with the effect of the plasma TCEA2 protein. However, the CKD risk was increased for TCEA2 gene expression in other tissues. The expression of GMPR in the brain and PLD3 in the blood was also consistent with the effects of the corresponding plasma proteins but showed an opposite effect in other tissues. Besides, although GATM and PFKFB2 also presented opposite effects across tissue-specific samples, the majority of the effects were protective and consistent with the effects of proteins. Therefore, a drug targeting these genes in different tissues may present potential off-target effects.

### Associations of protein with other CKD, kidney function, and CKD subtypes

**Fig. 4:**
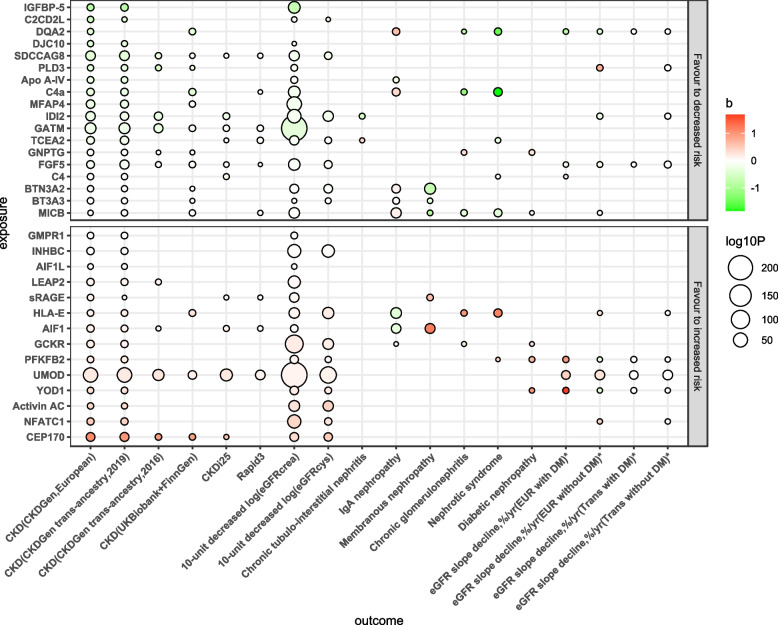
Balloon plot of identified proteins associated with extensive CKD-related phenotypes. The direction (increased or decreased risk) was determined by the estimators in the primary analysis for CKD (CKDGen, European). The color represents the* β* estimators of MR analysis, where green represents a decreased risk and red represents an increased risk for per-SD increased proteins. *The effects were adjusted and corresponded to increased risk (declined eGFR slope) and decreased risk (increased eGFR slope). EUR, European participants; Trans, trans-ancestry; DM, diabetes mellitus

**Fig. 5:**
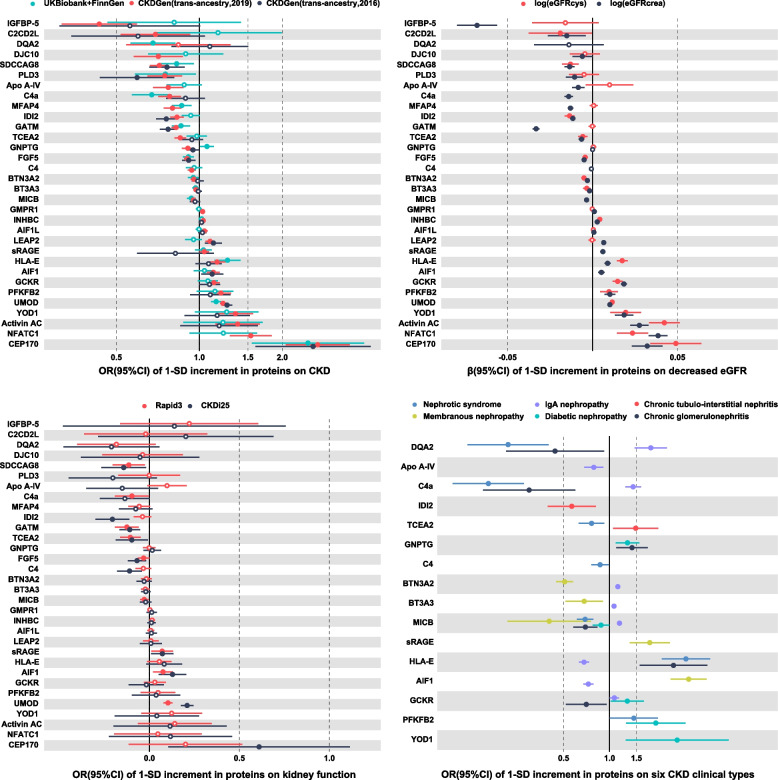
Associations of the 32 identified proteins with different CKD data sources, kidney function phenotypes, rapid kidney function decline phenotypes, and CKD clinical types. **A** Forest plot of the effect of 32 proteins on the risk of 3 additional CKD outcomes (data CKD2–4). **B** Forest plot of the effect of 32 proteins on 2 kidney function (eGFR) outcomes. **C** Forest plot of the effect of 32 proteins on the risk of 2 rapid kidney function decline outcomes. **D** Forest plot of the effect of these proteins on the risk of 6 CKD clinical types (only significant results with *P* < 0.05 were shown). Hollow dots represent *P* > 0.05, and solid dots represent *P* < 0.05

A summary of the associations of the 32 putative proteins with these outcomes is shown in Fig. 4, and the detailed results are shown in Fig. 5. All of the associations of identified proteins were replicated in the trans-ancestry CKD data, except for DQA2. In addition, 10 proteins were replicated in an earlier version of CKD data from CKDGen, and 14 proteins were replicated in the dataset from the UK Biobank plus FinnGen. For kidney function, 29 of 32 proteins were significantly associated with the eGFRcrea, except for DQA2, GNPTG, and C4. Moreover, 7 proteins were not significantly associated with the eGFRcys, in contrast to the eGFRcrea (Fig. 5). For rapid kidney function decline, SDCCAG8, GATM, TCEA2, and FGF5 were negatively associated with both CKDi25 and Rapid3, while sRAGE, AIF1, and UMOD were positively associated with both CKDi25 and Rapid3. For the clinical types of CKD, only IDI2 was significantly associated with a decreased risk of chronic tubulointerstitial nephritis. We found that Apo A-IV, HLA-E, and AIF1 were negatively associated with IgA nephropathy; however, HLA-E and AIF1 were positively associated with other CKD types. In addition, we observed that BTN3A2, BTN3A3, and MICB decreased the risk of membranous nephropathy, but sRAGE and AIF1 increased this risk. Additionally, DQA2, C4a, and MICB were protectively associated with both chronic glomerulonephritis and nephrotic syndrome. The effects of 32 proteins on annualized relative slope change of eGFR are shown in Additional file 1: Fig. S7. The DQA2, FGF5, IDI2 (for participants without diabetes), and MICB (for Europeans without diabetes) were positively associated with annualized eGFR slope change (represented decreased risk), while UMOD, HLA-E (without diabetes), NFATC1 (without diabetes), PFKFB2 (with diabetes), and YOD1 (with diabetes) were negatively associated with the slope change (represented increased risk), which were consistent with the principal findings. The effects of the 32 proteins on different outcomes are shown in Additional file 2: Table S7.

### Colocalization of the putative proteins with CKD

Among these proteins, NFATC1, PFKFB2, SDCCAG8, YOD1, FGF5, C2CD2L, sRAGE, GCKR, DJC10, Apo A-IV, TCEA2, IGFBP-5, and C4a were colocalized with CKD (PPH_4_ > 0.8), while BTN3A2, INHBC, MFAP4, BTN3A3, GNPTG, and activin AC were moderately colocalized with CKD (PPH_4_ > 0.5) (Additional file 1: Fig. S8, Additional file 2: Table S8).

### PPI network of putative proteins, Gene Ontology enrichment, and single-cell enrichment

**Fig. 6:**
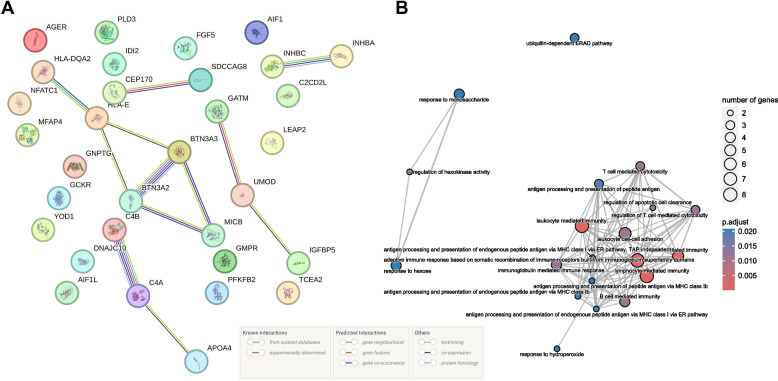
Results of protein–protein interaction network (**A**) and Gene Ontology enrichment pathways (**B**). For Gene Ontology enrichment pathways, only the top 20 GO terms are shown

As shown in Fig. 6A, HLA-DQA2, HLA-E, BTN3A2, BTN3A3, and MICB interacted with each other. Meanwhile, UMOD interacted with IGFBP-5 and GATM, and C4a interacted with C4b and Apo A-IV. In addition, CEP170 and SDCCAG8, INHBC, and INHBA (activin AC) also interacted with each other. Figure 6B presents the biological pathways of the significant genes. These genes were mainly enriched in T cell-mediated immunity, leukocyte-mediated immunity, lymphocyte-mediated immunity, adaptive immune response, and amide binding. In single-cell enrichment, FGF5, IGFBP-5, GATM, AIF1L, and UMOD mRNA presented kidney single-cell type enrichment, FGF5, C4a, GATM, PFKFB2, MFAP4, and UMOD mRNA presented kidney tissue cell type enrichment, while GATM, PFKFB2, MFAP4, PLD3, and AIF1 mRNA presented immune cell specificity. The full GO term and single-cell enrichment results for the corresponding genes are shown in Additional file 2: Tables S9-S10.

### Summary of the findings

Table 1 and Additional file 2: Table S11 summarize the findings of this study. According to the MR, SMR, and colocalization analysis, the 32 proteins that were causally associated with CKD and repeated for at least 2 CKD-related phenotypes were divided into 3 tiers. Tier 1 included 8 proteins that passed MR, SMR, and the colocalization analysis. Compared with previous studies, 11 of 32 loci (mapped genes) were identified by previous GWAS, 8 of 32 genes were identified by previous MR studies, and 6 of 32 proteins were identified by previous MR studies. The direction of the relationships between each protein and gene expression and CKD or related phenotypes in this study were consistent with previous MR evidence. In observational studies, 10 proteins were previously reported, of which sRAGE, LEAP2, AIF1, and PFKFB2 were consistent with observational evidence, while C4a, C4b, and UMOD were only consistent with previous MR. In summary, 20 proteins/coding genes were not reported by either previous transcriptome-wide or proteome-wide MR, and are novel causal findings.

**Table 1:** Summary of the findings

**Proteins (genes)**^a^	**Full name of the proteins**	**Direction for CKD risk of proteins**^b^	**Direction for CKD risk of genes**^b^	**No. of protein data sources**^c^	**Total repeated times**^**d**^	**Passed SMR analysis**	**Passed Coloc analysis**	**Druggable gene**^**e**^	**PPI**^**f**^	**Loci (gene) reported by GWAS**^**g**^	**Gene expression reported by MR**^**g**^	**Protein reported by MR**^**g**^	**Reported by observational study**^**g**^
**Tier 1**													
**FGF5**	**Fibroblast growth factor 5**	** − **	** − **	**1**	**11**	**Yes**	**Yes**	**Yes**	** − **	**Yes**	**Yes**	**Yes**	** − **
**C4a**	**Complement component 4A**	** − **	** − **	**1**	**6**	**Yes**	**Yes**	**Yes**	**Yes**	** − **	**Yes**	** − **	**Yes**
**BTN3A2**	**Butyrophilin subfamily 3 member A2**	** − **	** − **	**1**	**5**	**Yes**	**Yes**	**Yes**	**Yes**	** − **	**Yes**	** − **	** − **
GCKR	Glucokinase regulatory protein	+	+	2	5	Yes	Yes	−	** − **	Yes	−	−	−
IGFBP-5	Insulin-like growth factor-binding protein 5	−	−	1	2	Yes	Yes	Yes	Yes	Yes	−	−	Yes
sRAGE (AGER)	Advanced glycosylation end product-specific receptor, soluble	+	+	1	5	Yes	Yes	Yes	−	−	−	−	Yes
GNPTG	*N*-acetylglucosamine-1-phosphotransferase subunit gamma	−	−	1	2	Yes	Yes	Yes	−	−	−	−	−
YOD1	Ubiquitin thioesterase OTU1	+	+	1	6	Yes	Yes	−	−	−	−	−	−
**Tier 2**													
**C4 (C4a|C4b)**	**Complement C4**	** − **	** − **	**1**	**3**	**Yes**	** − **	**Yes**	**Yes**	** − **	**Yes**	** − **	**Yes**
**CEP170**	**Centrosomal protein of 170 kDa**	** + **	** + **	**1**	**6**	**Yes**	** − **	** − **	**Yes**	**Yes**	**Yes**	** − **	** − **
**IDI2**	**Isopentenyl-diphosphate delta-isomerase 2**	** − **	** − **	**3**	**8**	**Yes**	** − **	** − **	** − **	**Yes**	** − **	**Yes**	** − **
**INHBC**	**Inhibin beta C chain**	** + **		**2**	**3**	** − **	**Yes**	**Yes**	**Yes**	**Yes**	** − **	**Yes**	** − **
**SDCCAG8**	**Serologically defined colon cancer antigen 8**	** − **	** + **	**1**	**7**	** − **	**Yes**	** − **	**Yes**	**Yes**	**Yes**	** − **	** − **
**BT3A3 (BTN3A3)**	**Butyrophilin subfamily 3 member A3**	** − **		**1**	**5**	** − **	**Yes**	**Yes**	**Yes**	** − **	** − **	**Yes**	** − **
GATM	Glycine amidinotransferase, mitochondrial	−	− / +	2	6	Yes	−	−	Yes	Yes	−	−	−
AIF1L	Allograft inflammatory factor 1-like	+	+	1	2	Yes	−	−	−	−	−	−	−
DQA2 (HLA-DQA2)	HLA class II histocompatibility antigen, DQ alpha 2 chain	−	−	1	7	Yes	−	Yes	Yes	−	−	−	−
PFKFB2	6-Phosphofructo-2-kinase/fructose-2,6-bisphosphatase 2	+	− / +	1	7	−	Yes	−	** − **	Yes	−	−	Yes
NFATC1	Nuclear factor of activated T cells, cytoplasmic 1	+	−	1	5	−	Yes	−	** − **	Yes	−	−	−
Activin AC (INHBA|INHBC)	Inhibin beta A chain:inhibin beta C chain heterodimer	+		1	3	−	Yes	Yes	Yes	−	−	−	−
Apo A-IV	Apolipoprotein A-IV	−		1	3	−	Yes	Yes	Yes	−	−	−	Yes
MFAP4	Microfibril-associated glycoprotein 4	−		3	3	−	Yes	Yes	−	−	−	−	Yes
DJC10 (DNAJC10)	DnaJ homolog subfamily C member 10	−	+	1	2	−	Yes	−	−	−	−	−	−
C2CD2L	C2 domain-containing protein 2-like	−	+	1	3	−	Yes	−	−	−	−	−	−
TCEA2	Transcription elongation factor A protein 2	−	− / +	2	6	−	Yes	−	−	−	−	−	−
**Tier 3**													
**UMOD**	**Uromodulin**	** + **		**1**	**11**	** − **	** − **	**Yes**	**Yes**	**Yes**	** − **	**Yes**	**Yes**
**MICB**	**MHC class I polypeptide-related sequence B**	** − **	** + **	**1**	**9**	** − **	** − **	** − **	**Yes**	** − **	**Yes**	**Yes**	** − **
**LEAP2**	**Liver-expressed antimicrobial peptide 2**	** + **		**2**	**3**	** − **	** − **	**Yes**	** − **	** − **	**Yes**	** − **	**Yes**
HLA-E	HLA class I histocompatibility antigen, alpha chain E	+		1	8	−	−	−	Yes	−	−	−	−
PLD3	Phospholipase D3	−	− / +	1	4	−	−	−	−	−	−	−	−
AIF1	Allograft inflammatory factor 1	+	−	2	6	−	−	−	−	−	−	−	Yes
GMPR1	GMP reductase 1	+	− / +	1	2	−	−	−	−	−	−	−	−

Tier 1 included the proteins (genes) that passed MR, SMR, and the colocalization analysis, and tier 2 included the proteins (genes) that passed the MR plus SMR or the colocalization, while tier 3 only passed the MR analysis. Proteins or genes reported by previous MR studies were marked with bold font. The additional summarized results are shown in Additional file 2: Table S11

^a^These 32 proteins found in this study and the corresponding coding genes (shown in brackets if the names are different)

^b^The “ − ” and “ + ” were used to indicate the direction of each protein on CKD, where “ − ” represented a negative association while “ + ” represented a positive association. The “ − / + ” represented the gene expression presenting different effects across different tissues. The effects of C4 were marked according to the C4a gene

^c^The number (range 1–3) of a specific protein for CKD1 (Fig. 1A) in principal MR analysis that is significant at *P* < 0.05 across three protein data sources

^d^The number of total replication times for the combined CKD2–4, four kidney function outcomes, four relative slope changes of eGFR, and six CKD clinical types that are significant at *P* < 0.05 and have the same direction with the principal analysis

^e^Whether the coding genes of each protein were druggable was acquired from the previous study (PMID: 28356508)

^f^Whether interacted with other proteins in protein–protein interaction (PPI) analysis

^g^The evidence was acquired from the literature review of related studies. The corresponding PMID and the consistency are shown in Additional file 2: Table S11

## Discussion

This study revealed 32 proteins that are associated with CKD, kidney function, or some CKD clinical types. Among the 32 proteins, 12 proteins or genes have been reported by previous MR studies, including FGF5 [9, 12, 44], C4a [12, 45], C4b [12, 45], BTN3A2 [12, 45], CEP170 [12], IDI2 [12], INHBC [9, 12], SDCCAG8 [12], BTN3A3 [12], UMOD [9, 46, 47], MICB [12, 48], and LEAP2 [44]. In addition, IGFBP-5 [6, 49, 50], GCKR [5–7], GATM [5, 6], PFKFB2 [5, 7], and NFATC1 [5–7] were identified by previous GWAS. Since uromodulin (UMOD) has the smallest *P* value and a known role in eGFR and kidney disease, the UMOD could effectively serve as a positive control for our signal-identifying approach [9]. Our study also provides additional evidence of transcriptome-wide associations or proteome-wide associations for 20 novel proteins or their corresponding coding genes. These 32 previous and novel proteins or genes may be potential drug targets or biomarkers of CKD and kidney function.

Compared with previous observational studies, soluble receptors for advanced glycation end-products (sRAGE), liver-expressed antimicrobial peptide 2 (LEAP2), and allograft inflammatory factor-1 (AIF1) were positively associated with CKD or decreased eGFR and were consistent with our findings. Among them, sRAGE was significantly higher in patients with CKD than in controls [51–53] and was associated with the development of CKD (OR 1.39; 95% CI 1.06–1.83) and end-stage renal disease (OR 1.97; 95% CI 1.47–2.64) [54]. sRAGE is a potential biomarker of inflammation and oxidative stress. When AGEs interact with their cell-bound receptor (RAGE), cell dysfunction is initiated by activating nuclear factor kappa-B (NF-κB), increasing the production and release of inflammatory cytokines and hastening to decrease kidney function in CKD patients [55, 56]. Fasting plasma LEAP2 levels were inversely associated with the eGFR [*β*(95% CI) − 0.34 (− 0.56 to − 0.12)] [57]. LEAP2 is primarily secreted by the liver and increases with greater body mass and insulin resistance in individuals with prediabetes and overweight or obesity; therefore, an elevated LEAP2 level might indicate increased metabolic risk [57]. The serum AIF1 concentration was independently correlated with the logarithm of urinary albumin excretion (*β* = 0.213, *P* = 0.0120) and with the eGFR (*β* =  − 0.286, *P* = 0.0011) [58]. Mechanistically, aldosterone may induce vascular calcification related to chronic renal failure via the AIF1 pathway [59]. In addition, our SMR analysis with GTEx data for 6-phosphofructo-2-kinase/fructose-2,6-bisphosphatase 2 (PFKFB2) was also consistent with a longitudinal data analysis in American Indians, which reported that the variation in PFKFB2 appears to reduce PFKFB2 expression in adipose and kidney tissues and thereby increase the risk for adiposity and diabetic nephropathy.

Complement component 4a (C4a), complement component 4b (C4b), and UMOD were partly consistent with the observational studies but were consistent in previous MR analyses. We found that C4a had robust protective effects on CKD, kidney function phenotypes, and several clinical CKD types. In terms of gene expression, we observed the opposite effect of C4a and C4b on CKD risk. The TWAS of Schlosser et al. supported our findings that C4a and C4b increased and decreased the eGFR, respectively [12]. In observational studies, the serum C3/C4 ratio (HR 0.63, 95% CI 0.5–0.9) was found to be an independent predictor of renal outcomes in IgA nephropathy patients [60], while C4 levels (HR 2.4, 95% CI 1.6–3.8) were significantly associated with a poor prognosis among patients with IgA nephropathy [61]. In addition, another study showed that the gene expression of C4a increased the risk of IgA nephropathy, which was also consistent with our evidence that protein C4a is associated with IgA nephropathy. This finding implies that the effect of C4 on IgA nephropathy may be partly driven by component C4a. However, the effects of C4a and C4b on other types of kidney disease and kidney function have not been verified. Previous studies only revealed that C4a levels were higher in patients with focal segmental glomerulosclerosis, suggesting that complement activation contributes to glomerular injury and sclerosis [62]. Meanwhile, C4b was also upregulated in CKD, atherosclerosis, and hypertension [63]. C4a and C4b are likely involved in the complement system activation via the classical pathway [64]. However, the exact roles and mechanisms of C4, C4a, and C4b in the development of different CKD clinical types remain to be explored. For UMOD, MR identified plasma UMOD as a causal biomarker of CKD (OR 1.30; 95% CI 1.25–1.35) [47], and urinary UMOD was also significantly associated with lower eGFR and greater odds of eGFR decline or CKD [46]. However, the results of these observational studies are controversial. Köttgen et al. reported higher UMOD level was associated with an increased CKD risk (OR 1.72; 95% CI 1.07–2.77) [65], but Chen et al. reported that a lower UMOD level at baseline was associated with a greater risk of subsequent kidney failure with replacement therapy [66].

For tier 1 proteins, butyrophilin subfamily 3 member A2 (BTN3A2) and member A3 (BTN3A3) were previously reported as a target gene for schizophrenia, anxiety, cancer, etc. [67–69]. In this study, these genes were identified as protective biomarkers of CKD and risk factors for IgA nephropathy. BTN3A2 and BTN3A2 may play key roles in related diseases, including the increased IgA nephropathy risk (*β* = 0.0832, *P* = 1.24 × 10^−11^) [45], decreased autoimmune disease risk (e.g., systemic lupus erythematosus, *β* =  − 0.256) [70], decreased type 1 diabetes risk (*β* =  − 0.269566, *P* = 1.34 × 10^−23^) [71], and inhibits clear cell renal cell carcinoma progression by regulating the ROS/MAPK pathway via interacting with RPS3A [72]. Our finding of glucokinase regulatory protein (GCKR) was also consistent with a previous study showing that GCKR variability may play a pathogenetic role in both type 2 diabetes and CKD [73]. In addition, we found that both insulin-like growth factor-binding protein 5 (IGFBP-5) and its gene expression decreased CKD risk. However, a cross-sectional study showed the opposite trend for eGFR (*β* =  − 0.02) [11], but the causal evidence was still lacking. As apoptosis proteins, IGFBP-5 is involved in kidney-related diseases, such as diabetes, focal segment-sclerosing nephritis, and CKD physiological processes [74]. Single-cell sequencing revealed that IGFBP-5 is highly expressed in the renal interstitial and is the most highly expressed in kidney vascular endothelial cells; thus, it is related to CKD [75, 76]. *N*-acetylglucosamine-1-phosphotransferase subunit gamma (GNPTG) and *N*-acetylglucosamine-1-phosphotransferase subunit gamma (YOD1) are classically associated with mucolipidosis II/III and cancer [77–81], respectively. Whether these genes are involved in the mechanism of CKD or kidney function remains to be further explored.

For other proteins/genes, the inhibin *β*C chain (INHBC) is a member of the transforming growth factor* β* family and may be involved in the regulation of profibrotic pathways [82, 83]. This was consistent with our findings of activin AC (coded by INHBC), which was also positively associated with increased CKD risk and decreased kidney function. For serologically defined colon cancer antigen 8 (SDCCAG8), recessive mutations in the SDCCAG8 gene can cause a nephronophthisis-related ciliopathy with Bardet-Biedl syndrome-like features [84], and SDCCAG8 appears to interact with APOL1 to modulate the risk for nondiabetic end-stage kidney disease [85]. For the nuclear factor of activated T cells, cytoplasmic 1 (NFATC1), we found that plasma NFATC1 was associated with increased CKD risk. NFATC1 may participate in the mechanism of tubulointerstitial inflammation [86]; moreover, TNF-stimulated free cholesterol-dependent apoptosis in renal podocytes is also mediated by NFATC1 [87], and suppressing NFAT signaling can ameliorate podocyte injury [88]. Moreover, the proteins Apo A-IV and MFAP4 were also associated with CKD, but observational studies revealed different effects [89–91]. For Apo A-IV, a previous study determined that TNF-α induced increased Apo A-IV protein expression, which was related to proinflammatory acute kidney injury in human kidney cells [92]. MFAP4 is involved in unilateral ureteral obstruction-induced renal fibrosis through the regulation of the NF-κB and TGF-β/Smad pathways [93]. Besides, MICB may promote the development and progression of diabetic nephropathy [48]. However, MR analysis of protein and SMR analysis of gene expression presented different results, although our SMR analysis was consistent with previous studies. Our study also revealed that IGFBP-5, GATM, and C4a were only associated with the eGFRcrea but not with the eGFRcys. Nevertheless, C4a was also significantly associated with some CKD clinical types that were not defined by the eGFRcrea. However, GATM may be related to creatinine production rather than kidney function since it encodes glycine amidinotransferase, an enzyme involved in creatine biosynthesis [94]. In addition, there is limited evidence for the roles of DQA2, CEP170, IDI2, DNAJC10, C2CD2L, TCEA2, HLA-E, PLD3, and GMPR1 in CKD and kidney function. Future studies are required to explore potential associations between the expression of these genes and proteins in CKD-related phenotypes.

In terms of clinical relevance, the protein targets identified in our study suggest potential intervention measures in immune-related pathways. For example, C4a is a target of the clinical drug “human immunoglobulin G,” which is used to treat immunodeficiency and a wide variety of autoimmune disorders. We found that this protein was negatively associated with chronic glomerulonephritis and nephrotic syndrome but was positively associated with IgA nephropathy. This suggests that human immunoglobulin G and other targets may be repositioned for specific types of CKD treatment but also have potential side effects on IgA nephropathy. According to the GO and single-cell enrichment analysis, 8 genes participated in immunity-mediated pathways, 18 genes presented immune cell specificity, and 8 genes presented RNA single/tissue cell type specificity. These findings may provide novel targets for potential immunotherapies or target therapy for kidney disease. Additionally, these protein–protein interactions may be used to support combination therapy involving multiple targets.

Our study has several advantages. First, we integrated the largest proteome and transcriptome datasets to provide consistent targets of proteins and coding genes, which contributed to the identification of potential drug targets for CKD treatment. Second, we repeated our findings for 18 CKD-related phenotypes, including different CKD data sources, different kidney function phenotypes, and different CKD clinical types, which provided an atlas of putative biomarkers. Third, we performed our research by a comprehensive pipeline including the MR, SMR, colocalization, PPI, gene enrichment analysis, and comparisons with previous evidence, which supplied wide-angle evidence and implicated new roles of these proteins and genes from different viewpoints. To our knowledge, this may be the largest and most comprehensive proteome- and transcriptome-wide MR analysis of drug targets for CKD-related phenotypes. Some limitations should also be noted. First, because of the limited number of pQTLs and eQTLs, many proteins or coding genes were not included in the analysis, limiting the identification of additional candidate targets and verification of identified proteins. In addition, considering the potential differences in tissue- and cell-specific eQTLs, kidney cell-specific instruments should be applied when available in further study. Second, some of the mechanisms underlying our findings related to novel proteins are still unclear and require further study to explore potential biological mechanisms. Third, MR inevitably suffers from unknown horizontal pleiotropy, even if appropriate methods and sensitivity analyses are performed. Fourth, MR only provides evidence of a causal association and needs to be confirmed by future experimental studies. On the basis of generalizability, our evidence was replicated with multiple outcomes, different protein data sources, and tissue-specific associations, but whether this evidence is effective in the population still needs to be confirmed by further studies. Additionally, in the absence of a suitable method to compare the power of different analyses, the levels of evidence were only assessed by the consistent results from different analyses.

## Conclusions

We found 32 CKD-related proteins and 20 novel proteins that are associated with CKD, kidney function, and several CKD clinical types. According to MR, SMR, and colocalization analysis, FGF5, C4a, BTN3A2, GCKR, IGFBP-5, sRAGE, GNPTG, and YOD1 were identified as priority proteins for CKD treatment. These proteins and coding genes were mainly enriched in immunity-related pathways and enriched in kidney tissues or cells.

### Supplementary Information

Additional file 1. Supplement Text and Fig. S1-S8.

Additional file 2: Table S1. Sample overlap between CKD2 and CKD3. Table S2. F-statistics of identified 32 proteins. Table S3. Results of MR-Egger test. Table S4. Results of *Q* test for heterogeneity of pQTLs. Table S5. Results of 32 proteins on the risk of CKD. Table S6. The pQTL-exposure and pQTL-outcome associations. Table S7. Results of 32 proteins on different outcomes. Table S8. Results of colocalization of the putative proteins with CKD. Table S9. GO terms of enrichment analysis. Table S10. The annotations of RNA single cell type specificity, RNA tissue cell type specificity, and immune cell specificity. Table S11. The summarization of the findings of this study.

## Data Availability

Our pQTL summary data were acquired from previously published studies and can be found in the supplemental materials of these studies (https://www.nature.com/articles/s41588-021-00978-w [16], https://www.science.org/doi/10.1126/science.abj1541?url_ver=Z39.88-2003&rfr_id=ori:rid:crossref.org&rfr_dat=cr_pub%20%200pubmed [17], and https://www.nature.com/articles/s41586-023-06592-6 [18]). The CKDGen consortium meta-analysis data, including the CKD and kidney function, were acquired from https://ckdgen.imbi.uni-freiburg.de/ [95]. The GWAS data for the annualized relative slope change of eGFR can be accessed from https://www.kp4cd.org/node/1229 [26]. The summary data of other CKD types were extracted from the MR Base and MRC IEU OpenGWAS platform (https://www.mrbase.org/ and https://gwas.mrcieu.ac.uk/) via the TwoSampleMR R package [96, 97]. The SMR-formatted eQTL summary data of GTEx (V8) [20], CAGE [21], Westra et al. [22], and PsychENCODE [23] were acquired from the Yang Lab website (https://yanglab.westlake.edu.cn/software/smr/#eQTLsummarydata). The eQTL summary data of eGTLGen are available at https://eqtlgen.org/phase1.html [19]. The entire GWAS summary statistics for all proteins used in the colocalization analysis are available at https://www.decode.com/summarydata/ [98]. The related analysis code and pQTL summary data can also be found in GitHub (https://github.com/ssccsssdu/CKD_Multi_Omics.git) [99].

## Abbreviations

CKD: Chronic kidney disease
MR: Mendelian randomization
SMR: Summary-based MR
PWAS: Proteome-wide association study
TWAS: Transcriptome-wide association study
eGFR: Estimated glomerular filtration rate regulation
PPI: Protein-protein interaction
IVW: Inverse variance weighted
HEIDI: Heterogeneity in dependent instruments
