# Supplementary material for: Identification of novel therapeutic targets for chronic kidney disease and kidney function by integrating multi-omics proteome with transcriptome
Source: Genome Med. 2024 Jun 19;16:84. doi: 10.1186/s13073-024-01356-x (PMC11186236; doi:10.1186/s13073-024-01356-x)
Supplement: Supplementary file 1 — Additional file 1. Supplement Text and Fig. S1-S8. [file 13073_2024_1356_MOESM1_ESM.docx]

Supplement Text: A literature review was performed for 1) the GWAS of CKD and kidney function, 2) the MR studies of proteome and transcriptome with CKD and kidney function, 3) the observational studies of proteome and transcriptome with CKD and kidney function, and additional exploration of identified proteins or genes. We searched these studies from the PubMed website until March 10, 2024 with the following strategies and keywords.

- The GWAS of CKD and kidney function:
- (CKD[Title/Abstract]) AND (GWAS[Title/Abstract])
- (CKD[Title/Abstract]) AND (genome-wide-association studies[Title/Abstract])
- (chronic kidney disease [Title/Abstract]) AND (GWAS[Title/Abstract])
- (chronic kidney disease [Title/Abstract]) AND (genome-wide-association studies[Title/Abstract])
- (kidney function[Title/Abstract]) AND (genome-wide-association studies[Title/Abstract])
- (kidney function[Title/Abstract]) AND (GWAS[Title/Abstract])
- (kidney failure[Title/Abstract]) AND (GWAS[Title/Abstract])
- The MR studies of proteome and transcriptome with CKD and kidney function:
- (CKD[Title/Abstract]) AND (Mendelian randomization[Title/Abstract])
- (chronic kidney disease[Title/Abstract]) AND (Mendelian randomization[Title/Abstract])
- (kidney function[Title/Abstract]) AND (Mendelian randomization[Title/Abstract])
- (eGFR[Title/Abstract]) AND (Mendelian randomization[Title/Abstract])
- (--the identified proteins and genes in this study--[Title/Abstract]) AND (Mendelian randomization[Title/Abstract])
- (chronic kidney disease[Title/Abstract]) AND (transcriptome-wide association studies[Title/Abstract])
- (chronic kidney disease[Title/Abstract]) AND (proteome-wide association studies[Title/Abstract])
- (kidney function[Title/Abstract]) AND (transcriptome-wide association studies[Title/Abstract])
- (kidney function[Title/Abstract]) AND (proteome-wide association studies[Title/Abstract])
- (eGFR[Title/Abstract]) AND (transcriptome-wide association studies[Title/Abstract])
- (eGFR[Title/Abstract]) AND (proteome-wide association studies[Title/Abstract])
- The observational studies of proteome and transcriptome with CKD and kidney function:
- (chronic kidney disease[Title/Abstract]) AND (--the identified proteins and genes in this study-- [Title/Abstract])
- (kidney function[Title/Abstract]) AND (--the identified proteins and genes in this study-- [Title/Abstract])
- (eGFR[Title/Abstract]) AND (--the identified proteins and genes in this study-- [Title/Abstract]）(kidney[Title/Abstract]) AND (--the identified proteins and genes in this study-- [Title/Abstract]）
- (renal[Title/Abstract]) AND (--the identified proteins and genes in this study-- [Title/Abstract]）
- Additional exploration of identified proteins or genes
- (--the identified proteins and genes in this study-- [Title/Abstract]）


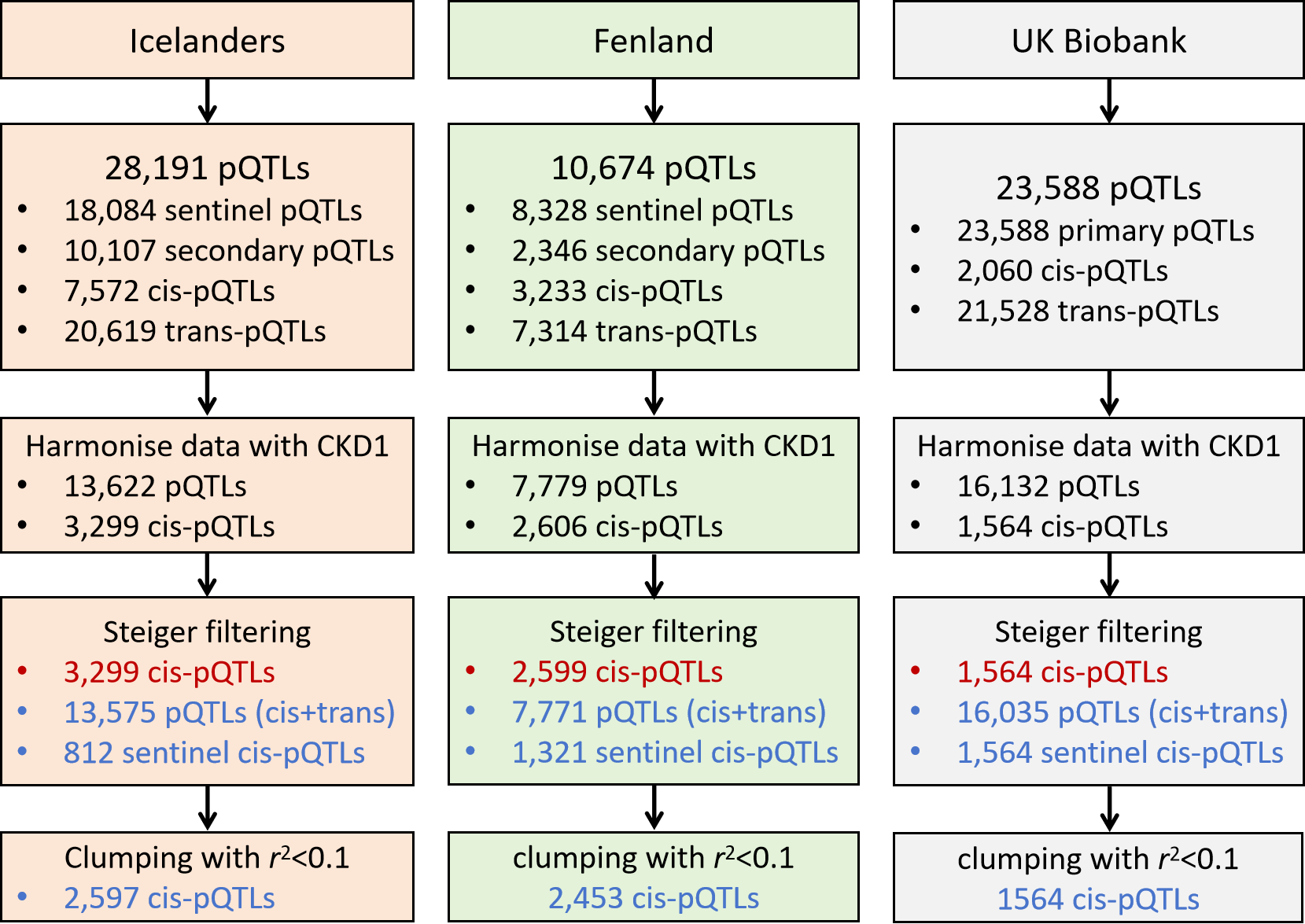


Fig. S1. The details of pQTLs in the three datasets.

The pQTLs marked with red were utilized in principal analysis and the pQTLs marked with blue were utilized in sensitivity analysis


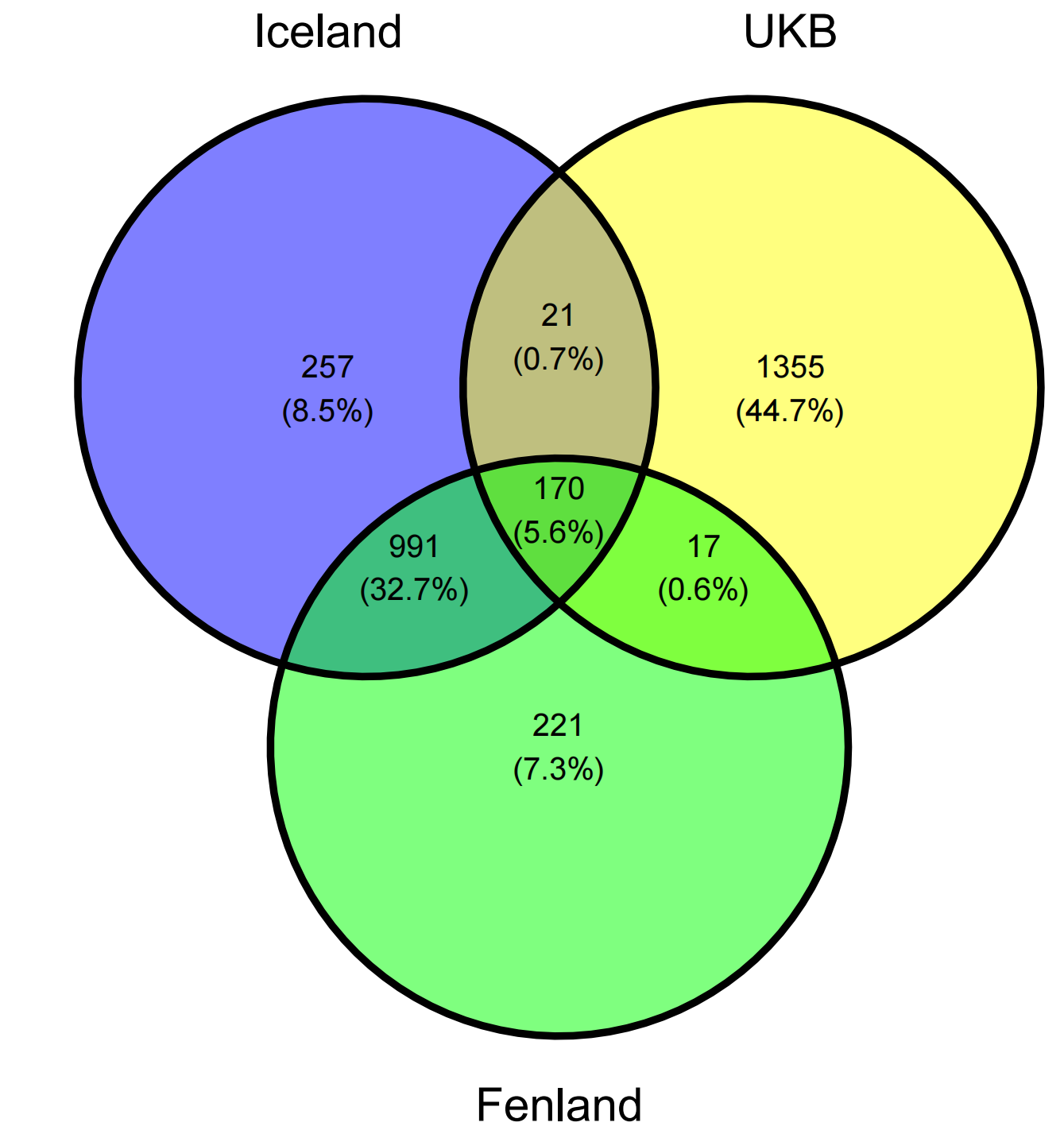


Fig. S2. Number and the overlap of proteins in the three datasets.


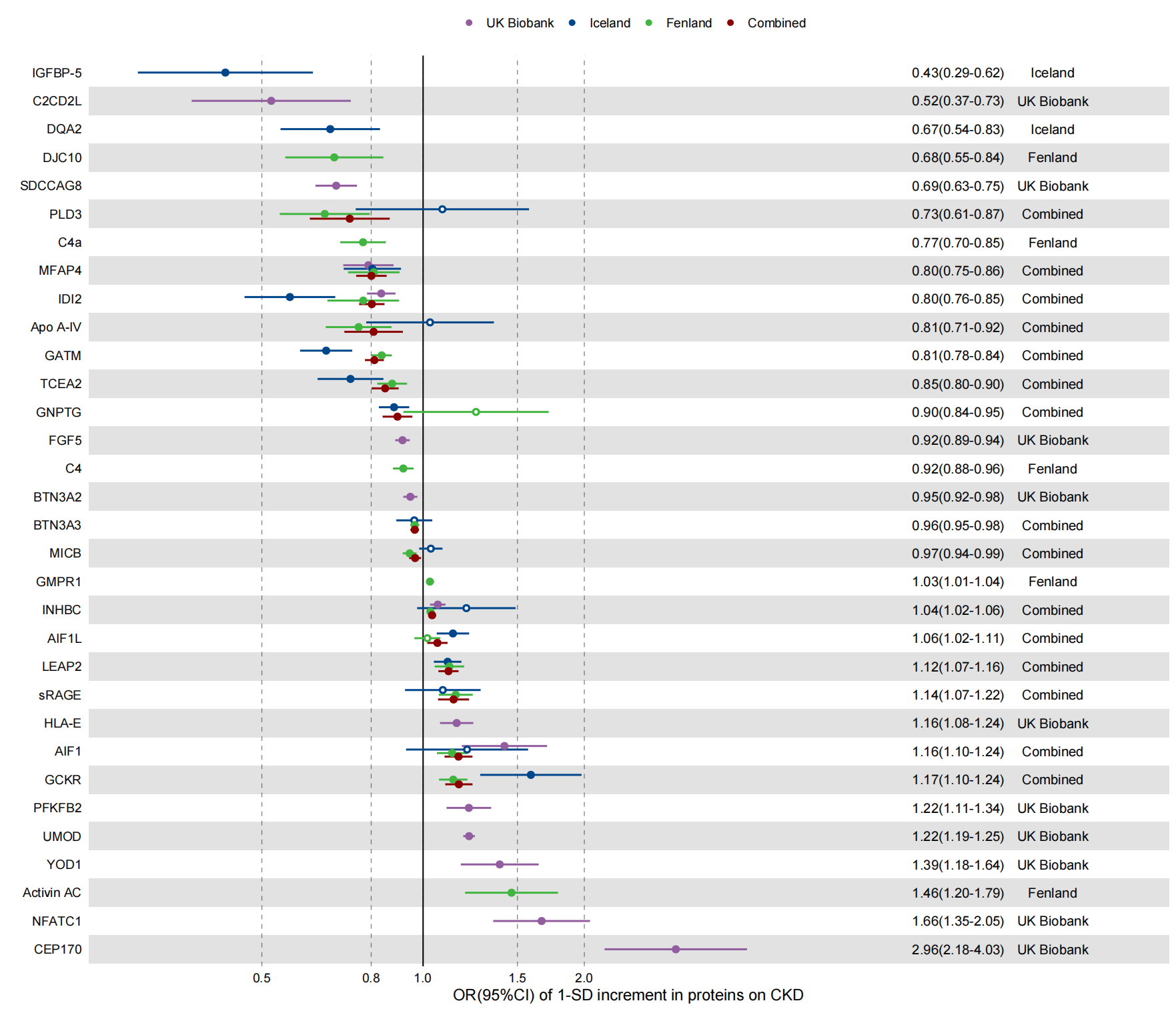


Fig. S3. The associations of 32 proteins with CKD by clumped pQTLs with *r*^2^<0.1.


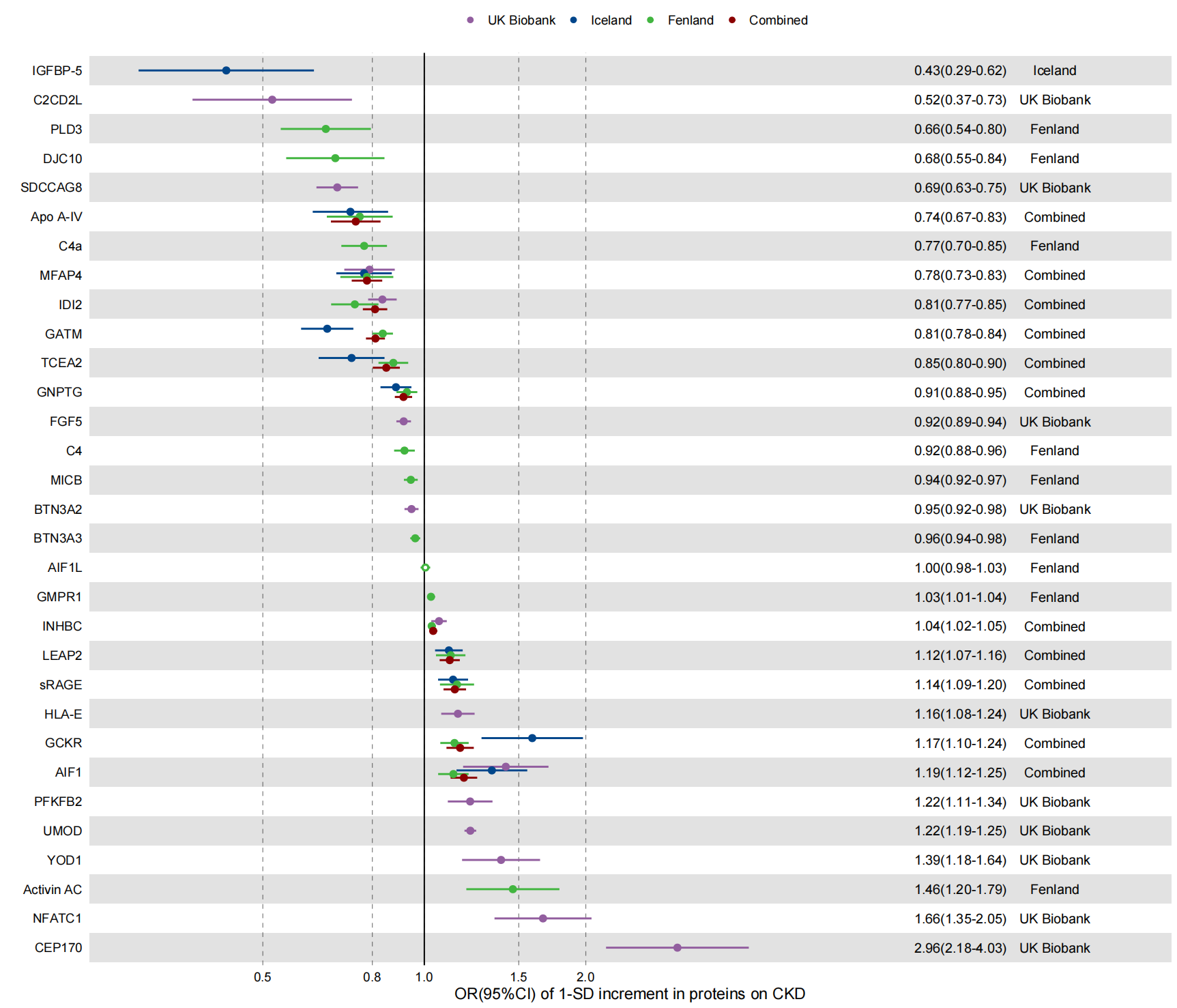


Fig. S4. The associations of 32 proteins with CKD by sentinel pQTLs.


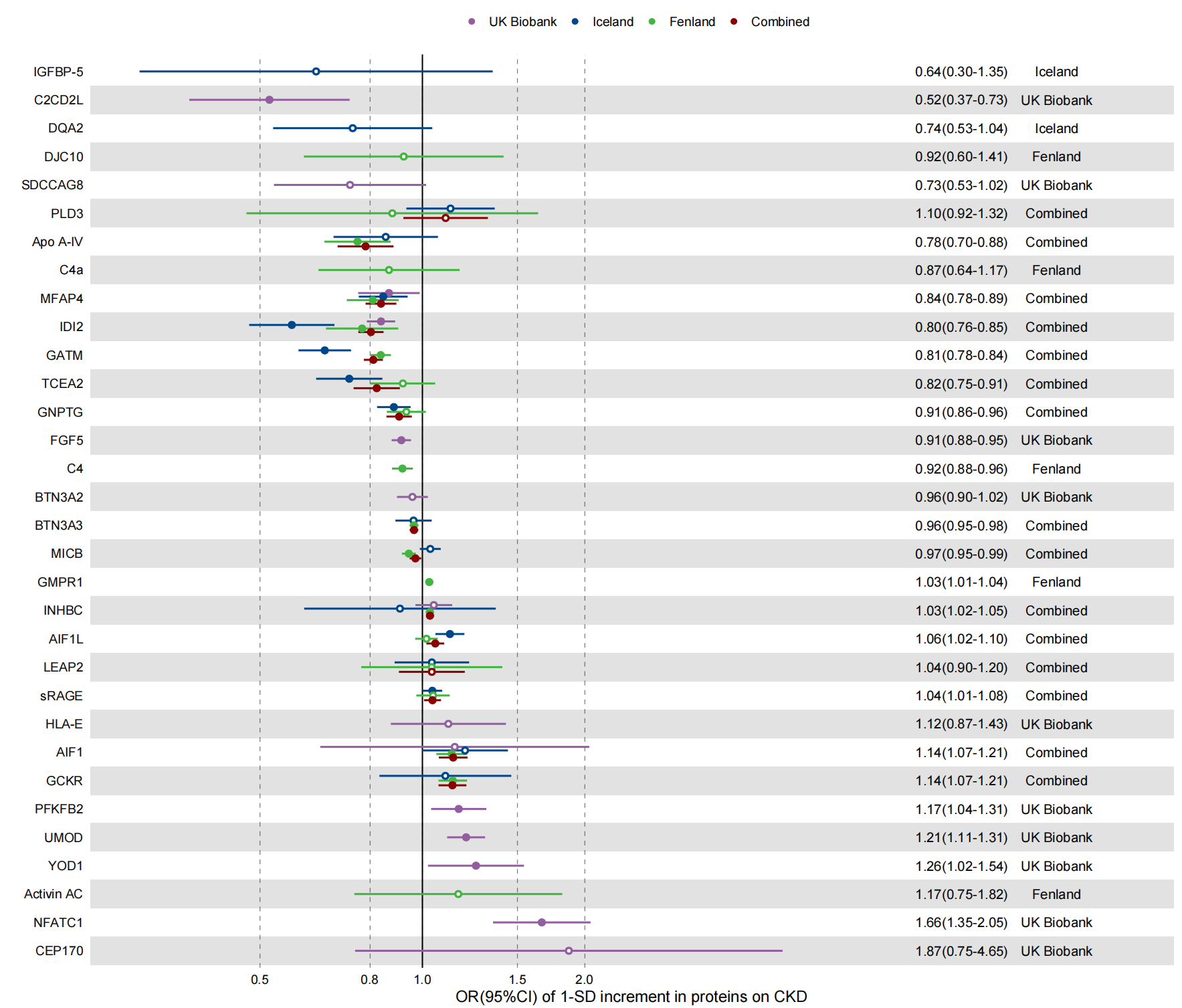


Fig. S5. The associations of 32 proteins with CKD by both cis- and trans-pQTLs.


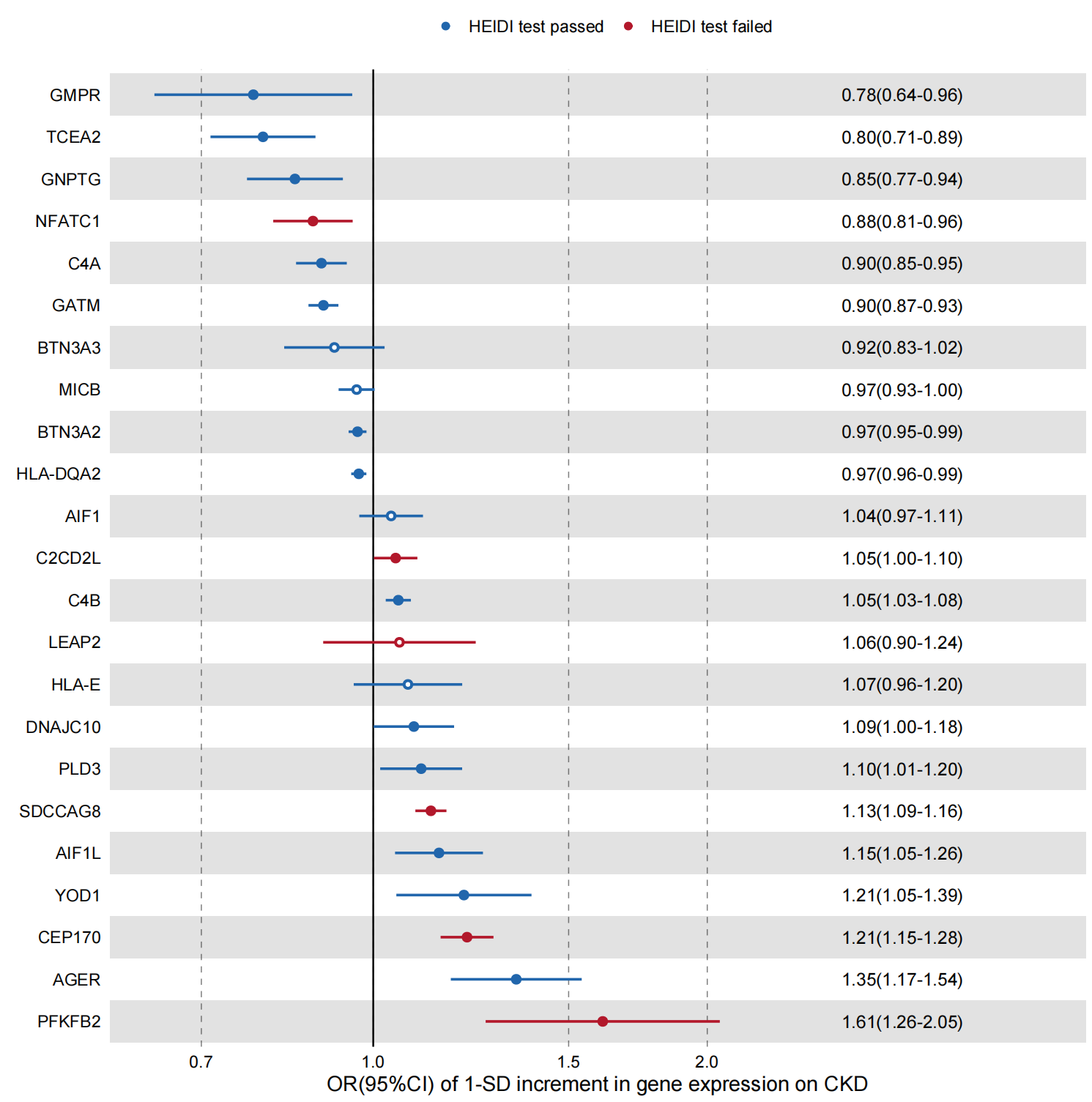


Fig. S6. ORs and 95%CIs of the mapped gene expression in the eQTLGen and CKD risk.


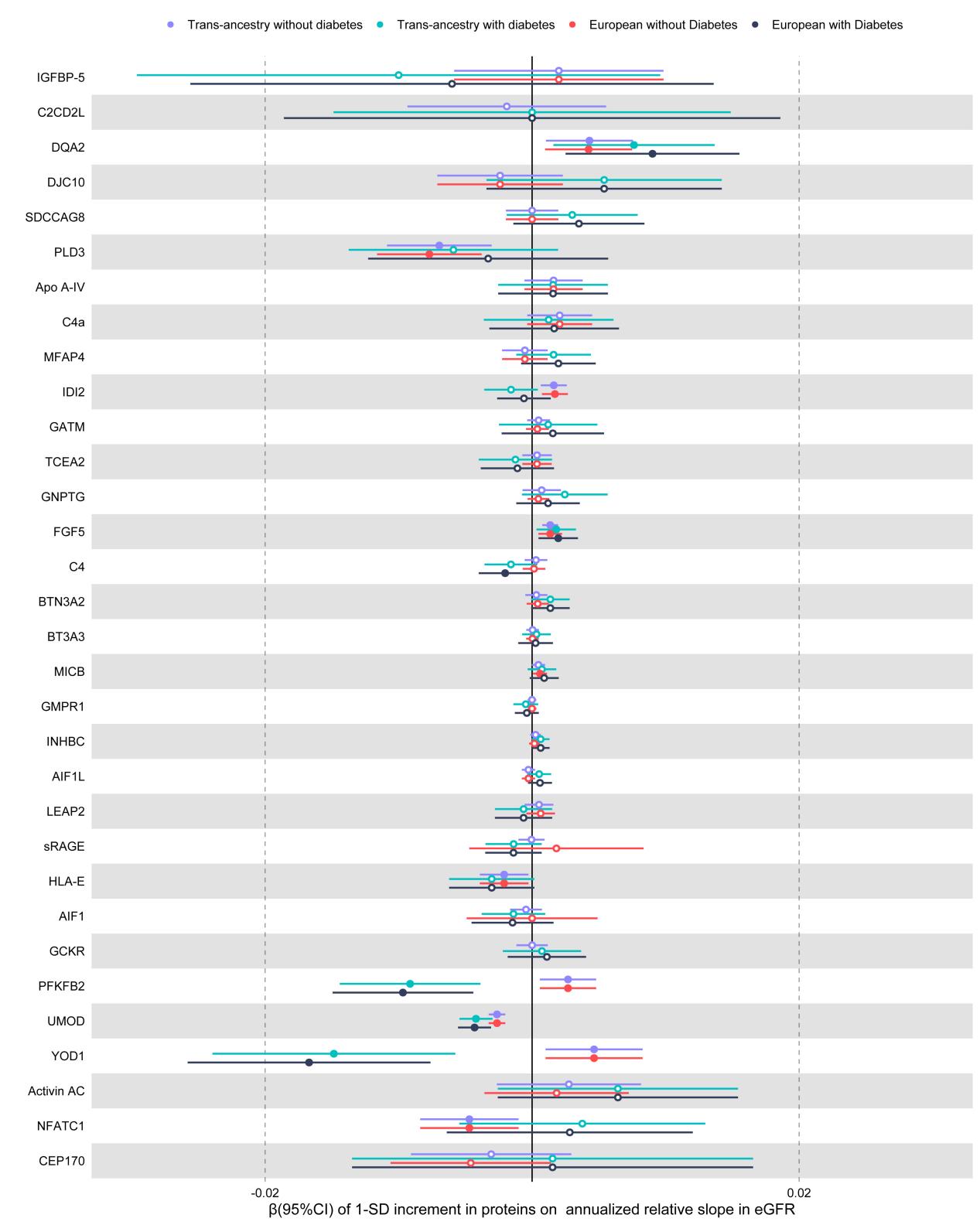


Fig. S7. Associations of the 32 identified proteins with annualized relative slope change.

Hollow dots represent *P*>0.05, solid dots represent *P*<0.05.

The association with increased slope indicates improved kidney function while the decreased slope indicates kidney function decline.


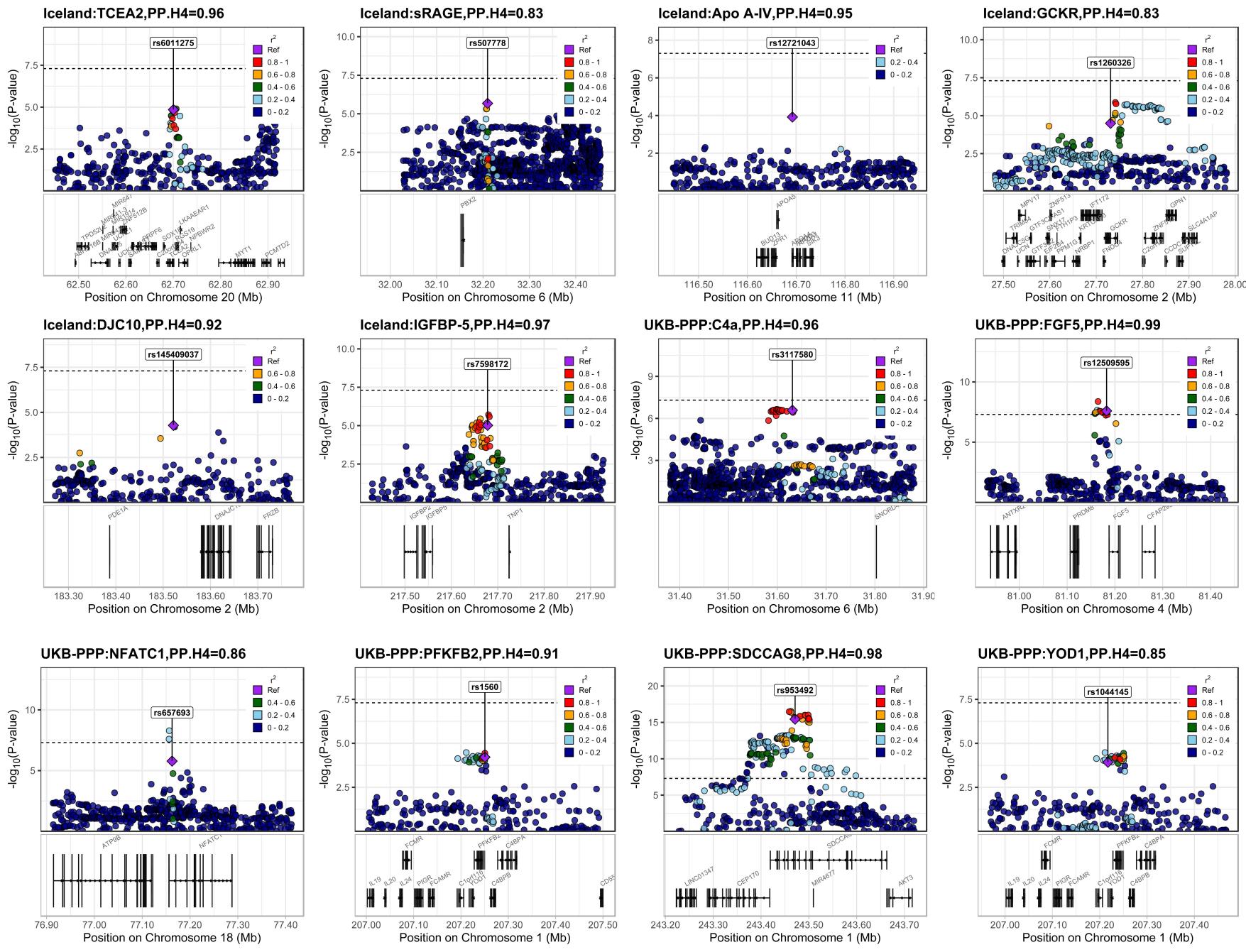


Fig. S8. The regional plot of colocalization analysis.

Only the cis-pQTL in Iceland and UKB-PPP with a PPH_4_>0.8 was shown. The full results were shown in Table S8.
